# 17‐a‐estradiol late in life extends lifespan in aging UM‐HET3 male mice; nicotinamide riboside and three other drugs do not affect lifespan in either sex

**DOI:** 10.1111/acel.13328

**Published:** 2021-03-31

**Authors:** David E. Harrison, Randy Strong, Peter Reifsnyder, Navasuja Kumar, Elizabeth Fernandez, Kevin Flurkey, Martin A. Javors, Marisa Lopez‐Cruzan, Francesca Macchiarini, James F. Nelson, Adrian Markewych, Alessandro Bitto, Amy L. Sindler, Gino Cortopassi, Kylie Kavanagh, Lin Leng, Richard Bucala, Nadia Rosenthal, Adam Salmon, Timothy M. Stearns, Molly Bogue, Richard A. Miller

**Affiliations:** ^1^ The Jackson Laboratory Bar Harbor ME USA; ^2^ Barshop Institute for Longevity and Aging Studies The University of Texas Health Science Center San Antonio TX USA; ^3^ Geriatric Research Education and Clinical Center San Antonio TX USA; ^4^ Research Service South Texas Veterans Health Care System San Antonio TX USA; ^5^ Department of Pharmacology The University of Texas Health Science Center San Antonio TX USA; ^6^ Department of Pathology and Geriatrics Center University of Michigan Ann Arbor MI USA; ^7^ Department of Psychiatry The University of Texas Health Science Center San Antonio TX USA; ^8^ Division of Aging Biology National Institute on Aging Bethesda MD USA; ^9^ Department of Physiology The University of Texas Health Science Center San Antonio TX USA; ^10^ Department of Pathology University of Washington Medical Center Seattle WA USA; ^11^ Department of Health & Human Physiology and Department of Biochemistry University of Iowa Iowa City IA USA; ^12^ Department of Molecular Biosciences University of California Davis CA USA; ^13^ Department of Pathology and Internal Medicine Wake Forest School of Medicine Winston‐Salem NC USA; ^14^ Department of Internal Medicine Yale University New Haven Connecticut USA; ^15^ Department of Molecular Medicine The University of Texas Health Science Center San Antonio TX USA

**Keywords:** 17‐α‐estradiol, candesartan cilexetil, geranylgeranylacetone, heterogeneous mice, lifespan, macrophage migration inhibitory factor, nicotinamide riboside

## Abstract

In genetically heterogeneous mice produced by the CByB6F1 x C3D2F1 cross, the “non‐feminizing” estrogen, 17‐α‐estradiol (17aE2), extended median male lifespan by 19% (*p* < 0.0001, log‐rank test) and 11% (*p* = 0.007) when fed at 14.4 ppm starting at 16 and 20 months, respectively. 90th percentile lifespans were extended 7% (*p* = 0.004, Wang–Allison test) and 5% (*p* = 0.17). Body weights were reduced about 20% after starting the 17aE2 diets. Four other interventions were tested in males and females: nicotinamide riboside, candesartan cilexetil, geranylgeranylacetone, and MIF098. Despite some data suggesting that nicotinamide riboside would be effective, neither it nor the other three increased lifespans significantly at the doses tested. The 17aE2 results confirm and extend our original reports, with very similar results when started at 16 months compared with mice started at 10 months of age in a prior study. The consistently large lifespan benefit in males, even when treatment is started late in life, may provide information on sex‐specific aspects of aging.

## Graphical abstract

Interventions that increase lifespan when started late in life are most likely to be useful in the clinic. Testing genetically diverse mice, the “non‐feminizing” estrogen, 17‐α‐estradiol, extended median male lifespan by 19% (*p* < 0.0001, log‐rank test) and 11% (*p* = 0.007) when fed at 14.4 ppm starting at 16 and 20 months, respectively. Nicotinamide riboside and three other drugs did not affect lifespan in either sex.

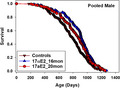

## 1 INTRODUCTION

Each year, the Interventions Testing Program (ITP; http://www.nia.nih.gov/research/dab/interventions‐testing‐program‐itp) tests the effects of a variety of compounds on lifespan in a genetically heterogeneous mouse model (UM‐HET3), the first‐generation offspring of the CByB6F1 x C3D2F1 cross, to produce a diverse heterogeneous population that is reproducible across time and place (Roderick, 1963). ITP studies are conducted simultaneously at The University of Texas (UT), University of Michigan (UM), and The Jackson Laboratory (TJL) in Bar Harbor, ME. Details of the ITP design have been published (Harrison et al., 2009; Harrison et al., 2014; Harrison et al., 2019; Macchiarini et al., 2020; Miller et al., 2011, 2014; Miller, Harrison, et al., 2020; Strong et al., 2008, 2016, 2020). Interventions that are found, in an initial experiment, to increase lifespan are then subsequently tested for physiological and pathological changes with age. The data in this paper come from the initial survival cohorts, and thus focus on lifespans, which integrate all biological effects that may lead to death. The spectrum of lethal pathology in UM‐HET3 mice has been tabulated by Lipman et al. (2004), using groups of 136 and 208 virgin females, 268 multiparous females, plus 117 and 157 virgin males.

The interventions for the present study were chosen for the following reasons:

(a) 17‐α‐estradiol (17aE2) is a relatively “non‐feminizing” estrogen which shows reduced activation of classical estrogen receptors compared with 17‐β‐estradiol (Anstead et al., 1997). Harrison et al. (2014) reported that in UM‐HET3 mice fed 4.8 mg 17aE2/kg (4.8 ppm) diet from 10 months of age, median male lifespans increased 12% (*p* = 0.0012, pooled across the three sites), while 17aE2 did not alter female lifespan. Strong et al. (2016) showed that using a threefold higher dose (14.4 ppm) from 10 months of age, pooled median male lifespans increased 19% (*p* < 0.001); the 90% lifespan increased 12%, but females still did not benefit. Thus, only males were tested in the present study. To determine whether 17aE2 treatment is effective when initiated in older mice, males were treated beginning at 16 or 20 months of age, choosing middle age, and early old age before many natural deaths.

(b) Nicotinamide riboside (NR) is a precursor of nicotinamide adenine dinucleotide (NAD) via the cell's salvage pathway (Trammell, Schmidt, et al. (2016)). Total NAD levels decline with age, in a wide range of species. Importantly, increasing NAD levels benefit a wide variety of tissues in species including mice and human beings. Rajman et al. (2018), for example, suggest that NAD+boosters may “..delay aging and age‐related physical decline.” Zhang et al. (2016) reported that NR delays senescence of neural SCs and melanocyte SCs and increases mouse life span, even when given in old age (5% increase at 20 months of age).

Trammell, Schmidt, et al. (2016) reported that in mice and humans NR is bioactive when given by mouth, unlike most other nicotinamide derivatives. In 2016b, they reported that NR improved liver function and protected against diabetic neuropathy. When fed to C57BL/6 J mice from 10 weeks of age, NR protects against high‐fat diet (HFD)‐induced obesity and promotes oxidative metabolism by increasing the NAD+/NADH ratio in muscle, liver, and brown adipose tissue (Canto´et al., 2012). Ryu et al. (2016) found that increasing NAD+stores with NR supplementation improved muscle function and alleviated heart defects in a mouse model of muscular dystrophy. Mitchell et al. (2018) reported that an NR metabolite, nicotinamide, did not increase lifespan when started at 12 months in C57BL/6 J mice but improved some health outcome measures. Due to its benefits in a variety of diseases, and reports of benefits in mouse lifespans, NR treatment was proposed to increase lifespan in UM‐HET3 mice.

c) Candesartan cilexetil (CC) is an angiotensin‐receptor blocker, which lowers blood pressure (Ikeda et al., 2006) and improves cardiovascular function and insulin sensitivity in obese, hypertensive patients (Grassi et al., 2003). Importantly, angiotensin‐receptor knockout increases lifespan of mice (Benigni et al. 2009). Because CC is effective against age‐related diseases, and sensitizes the body to insulin, and because the angiotensin‐receptor knockout increases lifespan of mice, treatment with CC was hypothesized to increase lifespan.

(d) To maintain good quality protein in the body, heat shock proteins (HSPs) are vital. Geranylgeranylacetone (GGA) induces heat shock protein (Hsp70) in mammalian tissues and promotes insulin sensitivity in old mice (Silverstein et al., 2015), while van Marion et al. (2020) showed that it increases HSP expression in atrial tissue after heart surgery. Pride et al. (2015) showed that long‐lived species, compared with related short‐lived species within the same order, have elevated HSP levels in conjunction with better proteostasis. To test whether treatment with an established HSP inducer can increase lifespan in a mammalian model, UM‐HET3 mice were treated with GGA.

(e) MIF098 ((3‐(3‐hydroxybenzyl)‐5‐methylbenzo[d]oxazol‐2(3H)‐one) is a macrophage migration inhibition factor (MIF) antagonist that regulates CD44 binding (Yoo et al., 2016). MIF is a proinflammatory cytokine, so MIF098 reduces inflammation. This may include the chronic inflammation that increases with age, as suggested by the finding of Harper et al. (2010) that MIF‐knockout mice live significantly longer than controls. Because it is orally bioavailable and shows MIF inhibitory activity in mouse models of hyperoxic lung injury, as well as in other diseases (Sauler et al., 2015), treatment with MIF098 was proposed to increase lifespan by decreasing chronic inflammation and disease (Poulsen et al., 2019).

Our new data show that Nicotinamide riboside (NR) failed to increase lifespan. Only 17aE2 increased lifespan, and benefits in males occurred even when the drug was not fed until late middle or early old age (16 and 20 months of age, respectively). The range of ages for which treatment is effective suggests that benefits from 17aE2 do not depend on effects earlier in life, such as growth alteration. Interventions that are effective when started at a late age have considerable translational potential.

## 2 RESULTS

**FIGURE 1:**
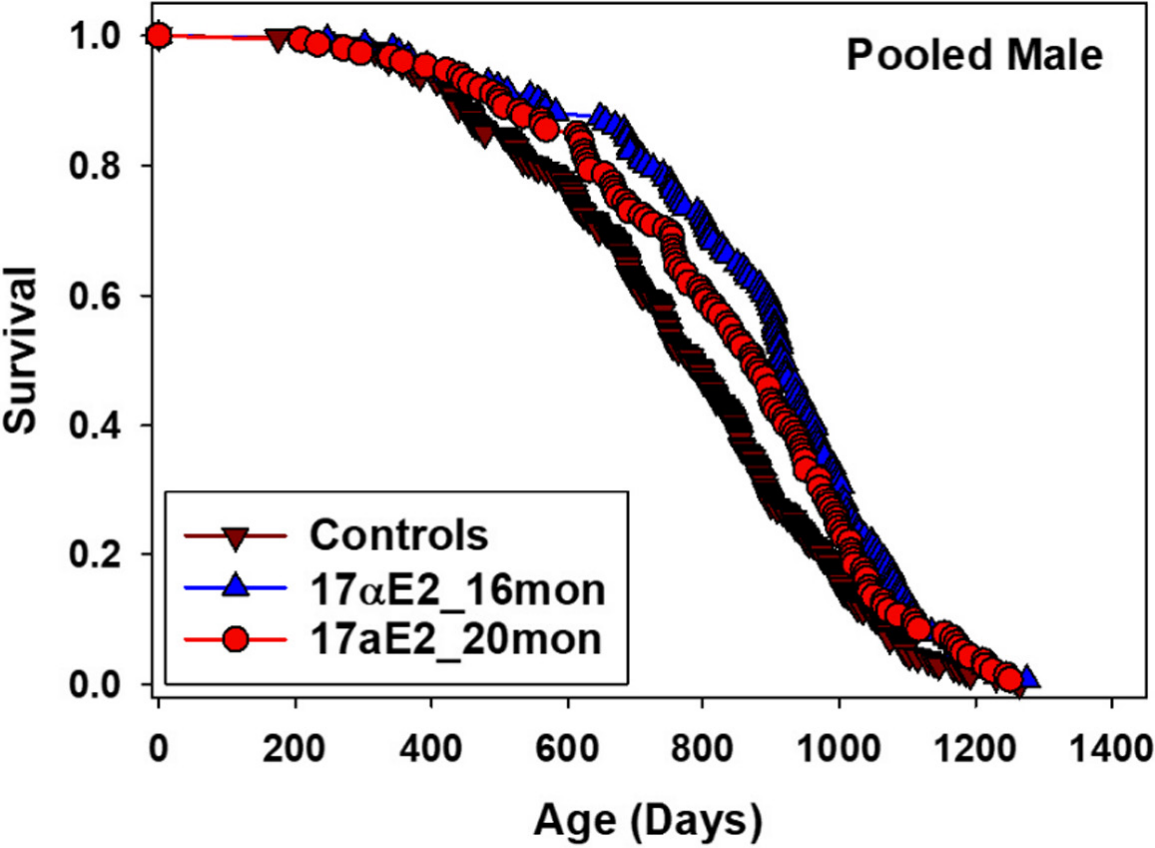
Effects of 17aE2 on lifespan curves showing male control and males fed 17aE2 diets started at 16 months or at 20 months of age. Table 1 gives more details on these lifespans

Compared to controls, UM‐HET3 male mice fed a diet with 14.4 ppm 17aE2 starting at 16 or 20 months of age had, respectively, 19% (*p* < 0.0001, log‐rank test) and 11% (*p* = 0.007) increases in median lifespans (data pooled across three sites). Lifespans at the 90th percentile were significantly increased 7% when treatment was started at 16 months (*p* = 0.004, Wang–Allison statistic); however, when treatment was started at 20 months, the 5% increase in the 90th percentile lifespan was not significant (*p* = 0.17) (Table 1; Figure 1). These data hint that benefits from 17aE2 diminish when treatment is started at 20 rather than at 16 months; however, when comparing the median lifespan results between the two treatment groups, no significant difference was detected (*p* = 0.24, log‐rank test).

**TABLE 1:** Effects of interventions on lifespan; data pooled from the 3 sites

		Median lifespan			Lifespan at 90th percentile		
Group	*N*	Days	% change	Log‐rank *p*‐value	Days	% change	Wang–Allison *p*‐value
Females							
Cont_16	304	874			1047		
NR	136	875	0%	0.612	1068	2%	0.99
CC	136	880	1%	0.235	1120	7%	0.092
GGA	136	894	2%	0.72	1081	3%	0.176
MIF098	136	863	−1%	0.069	1028	–2%	0.314
Males							
Cont_16	303	787			1047		
17aE2 16	156	933	19%	<0.0001	1120	7%	0.004
17aE2 20	159	871	11%	0.007	1103	5%	0.174
NR	156	763	−3%	0.252	1019	−3%	0.99
CC	156	820	4%	0.370	1055	1%	0.733
GGA	156	825	5%	0.720	1070	2%	0.730
MIF098	156	821	4%	0.645	1055	1%	0.99

Lifespans of ITP mice from cohort 2016.

Group: Cont_16 fed control diet; 17aE2 16 and 20 fed 14.4 ppm 17‐α‐estradiol starting at 16 and 20 months of age. Mice started on diets at 8 months of age were those fed: NR with 1,000 ppm nicotinamide riboside, CC with 30 ppm candesartan cilexetil, and MIF098 with 240 ppm MIF098. GGA mice were fed 600 ppm geranylgeranylacetone starting at 9 months of age.

*N* = number of mice tested; data were pooled, with about 1/3 of the mice from each testing site; see Table 2 caption for details.

Under Median lifespan: Days—median ages; % change calculated with respect to controls. *p*‐value = probability that lifespans are the same as the controls using two‐tailed log‐rank test on pooled data stratified by sites; "removed" mice were included as censored (see Experimental Procedures).

Under lifespan at 90th percentile: Days = age at 90th percentile, % change from control.

Wang–Allison *p*‐value = probability that the proportion of live mice is the same in treated as in the control group at the 90th percentile age, evaluated by the procedure of Wang et al. (2004).

**FIGURE 2:**
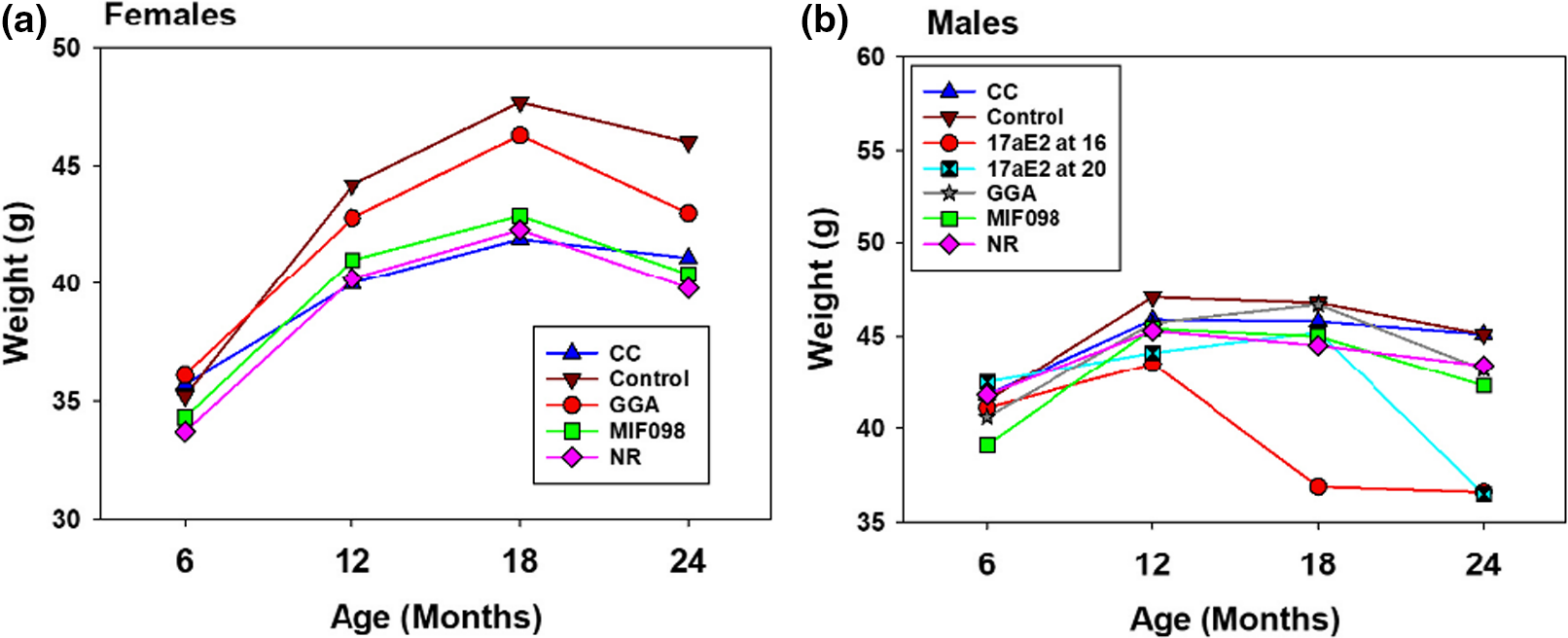
Effects of interventions on body weights. Panel A shows females, and Panel B shows males. Weights are from the same mice whose lifespans are shown in Table 1. Control mice were fed the control diet; 17aE2 started at 16 or 20 months of age. CC, MIF098, and NR were fed from 8 months of age, while GGA was fed from 9 months of age

Mice were weighed every 6 months beginning at 6 months of age. Prior to treatment, at 6 and 12 months of age, the mean weight of each group of mice designated for treatment was comparable to the weight of the group designated as the untreated control group. Weights of male mice started on 17aE2 at 16 months decreased about 20% by 18 months, and weights of mice started on 17aE2 at 20 months decreased about 20% by 24 months (Figure 2B). At 24 months, the weights of both of the 17aE2 treatment groups were similar, and both were about 8.5 g lighter than controls (*p* < 0.001 in both cases).

Comparing median lifespans across individual sites (Table 2), starting males on 17aE2 diets at 16 months increased lifespans 20% at both TJL (*p* = 0.001, log‐rank test) and UT (*p* < 0.0001), with a 16% increase at UM (*p* = 0.06). Starting 17aE2 diets at 20 months increased lifespans 17% at TJL, 11% at UM, and 5% at UT; only the 17% increase was significant (*p* = 0.02). Comparing median lifespans within each site, starting 17aE2 treatment at 16 months tended to increase lifespans more than starting treatment at 20 months (Table 2), although the difference reached significance only at UT (*p* = 0.003).

**TABLE 2:** Effects of Est‐16 and NR on median lifespan at each ITP site

Group	TJL			UM			UT			Mean % change
	Days	% change	*p*‐value	Days	% change	*p*‐value	Days	% change	*p*‐value	
Females										
Cont_16	883			858			882			
NR	968	10%	0.040	799	−7%	0.033	857	−3%	0.335	0%
CC	896	1%	0.124	867	1%	0.371	876	−1%	0.413	0%
GGA	905	2%	0.461	916	7%	0.152	892	1%	0.930	3%
MIF098	895	1%	0.874	836	−3%	0.084	866	−2%	0.347	−1%
Males										
Cont_16	752			826			799			
17aE2 16	906	20%	0.006	943	14%	0.110	955	20%	0.0001	18%
17aE2 20	896	19%	0.027	900	9%	0.160	840	5%	0.444	11%
NR	757	1%	0.968	852	3%	0.398	712	−11%	0.019	−3%
CC	826	10%	0.035	866	5%	0.487	770	−4%	0.689	4%
GGA	787	5%	0.729	914	11%	0.156	801	0	0.875	5%
MIF098	829	10%	0.228	810	−2%	0.721	826	3	0.491	4%

For each site, TJL (the Jackson Laboratory), UM (University of Michigan), UT (University of Texas), numbers in treated groups were 51–54 per site for males and 44–48 for females; numbers of controls were 99–102 for males and 92–116 for females.

This table lists: Group—the same as Table 1. Days = median survival in days, % change from control, and the log‐rank P that the group differs from the controls (Cont_16). The rightmost column shows the average (mean) of the changes in median lifespan across the three sites.

**FIGURE 3:**
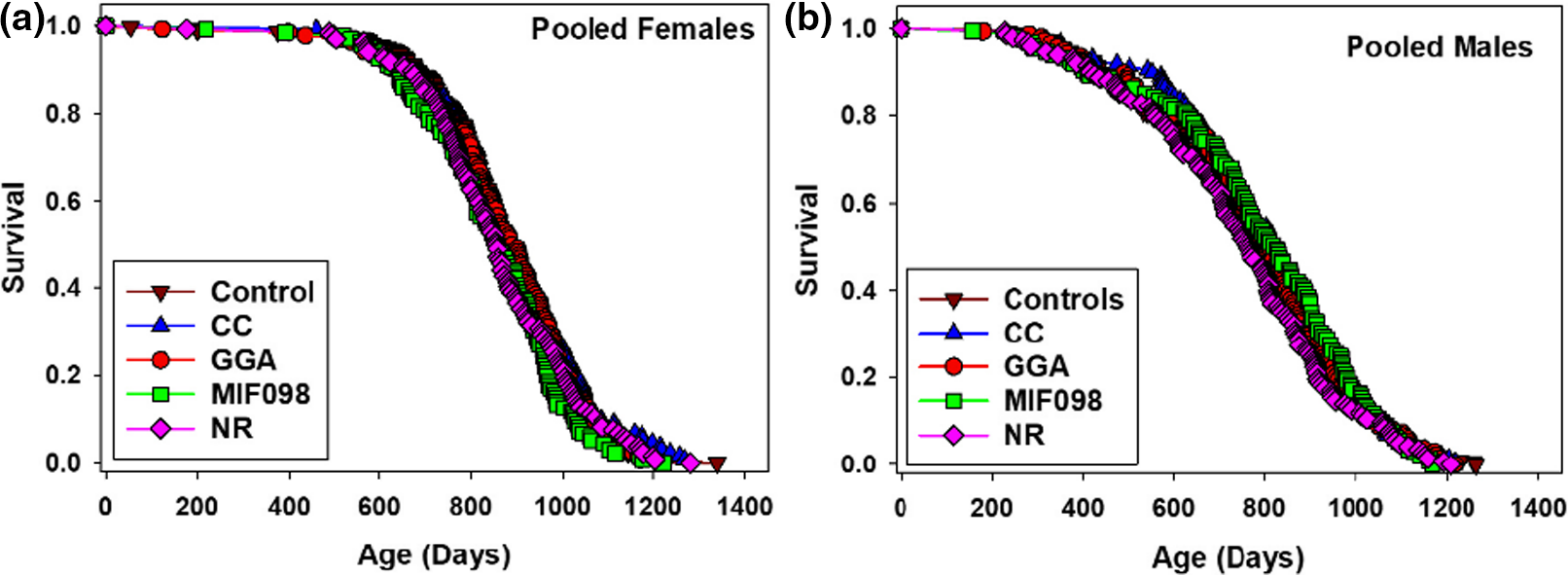
Lifespan curves of controls, and the 4 interventions without effects/. Panel A shows females, and Panel B shows males. Data are from the same mice whose lifespans are shown in Table 1

Lifespans for pooled data (across the three sites) were not significantly affected in UM‐HET3 males or females fed diets with 1,000 ppm nicotinamide riboside (NR), 30 ppm candesartan cilexetil (CC), 600 ppm geranylgeranylacetone (GGA), or 240 ppm MIF098 (Table 1, Figure 3). Site‐specific effects on lifespans were also not significant, although the site‐specific data sets had far less statistical power (Table 2). NR led to a marginally significant increase in female lifespan at one site, but this was balanced by a marginally significant decline in female lifespan at another (as well as a marginally significant decline in lifespans of males at a single site) (Table 2). The ITP primary endpoint is always the pooled data set, and NR had no significant effect, positive or negative, in the pooled data sets for either sex. Figure 2 gives effects of these agents on body weights. In females, weights of mice fed GGA were similar to weights in controls, while females fed NR, CC, and MIF098 were about 5 g lighter than controls. In males, weights of mice fed NR, CC, GG, and MIF098 were similar to weights in controls.

As detailed in the Experimental Procedures, NR, CC, GGA, and MIF098 were each present in the diet pellets at about the amounts expected. Nicotinamide was detected in serum at the following levels (mean ± *SD* [*n*], ng/ml): controls 105 ± 46 (*n* = 19) and NR‐treated 401 ± 212 (*n* = 31), with males and females pooled because their results were similar. Treated mice had significantly more nicotinamide (*p* < 0.0001 by Mann–Whitney test). No NR was detected, but the detection limit was 500 ng/ml, while that of nicotinamide was 25 ng/ml. Possibly, by the time NR was in the blood, it had already been metabolized to nicotinamide.

In a separate study, NR and its metabolites were tested in liver, brain cortex, plasma, gastrocnemius muscle, heart, kidney, iWAT (inguinal white adipose tissue), gWAT (perigonadal white adipose tissue), and leg muscle from 6‐ to 10‐month‐old UM‐HET3 mice fed control or 1,000 ppm NR diet for 6–6.5 weeks, with 3 males and 4 females per group. The most interesting positive finding was lower levels (*p* = 0.004) of nicotinamide in cortex (though not liver) of NR‐treated mice. NR‐treated mice also had lower NR levels in cortex (but not in liver), compared with controls (*p* = 0.03). The ratio of NAD to NADH in the cortex was lower in NR‐treated males (*p* = 0.012). These results are preliminary, because numbers of mice tested were low, and the diets were only fed to young mice for 6 weeks.

Candesartan (but not CC) was found in plasma of all four female and three male UM‐HET3 mice fed CC for 8 weeks: values (ng/ml) for the females were 25, 44, 19, and 49; values for the males were 40, 26, and 23. The four female and three male controls all had less than the 5 ng/ml detection limit of CC. In mice receiving dietary GGA and MIF098, amounts were below detection limits in serum (1 µg/ml and 15 ng/ml, respectively).

## 3 DISCUSSION

Earlier ITP studies found that 17aE2 treatment, initiated at 10 months of age, increased lifespan in UM‐HET3 males but had no effect on UM‐HET3 females. To determine whether later onset of 17aE2 treatment is comparably effective, we evaluated UM‐HET3 males treated with 17aE2 initiated at either 16 or 20 months of age, roughly equivalent to humans of 50 and 60 years of age. Lifespan was significantly increased in both treatment groups compared with controls. Although the increased lifespans of the males started on the 17aE2 diets at 16 or 20 months were not statistically different (log‐rank test), males that started treatment at 16 months tended to live longer, as indicated both by pooled data (Table 1, Figure 1) and site‐specific data (Table 2). Providing a strong test of this impression would require higher numbers of animals.

A feature of the ITP design is that husbandry conditions and derivation of the mice are standardized across sites and time; thus, results collected in different ITP studies of the same treatments are highly comparable. In an earlier study from the ITP, Strong et al. (2016) found a 19% increase in lifespan when mice were given 14.4 ppm 17aE2 from 10 months of age (17aE2‐fed, 925 days; controls, 780 days). In the current study, median lifespans were similar, despite being collected 5 years later and starting the 17aE2 diet at 16 rather than 10 months of age (17aE2‐fed, 933 days; controls, 787 days) (Table 1). Results for lifespan at the 90th percentile differed slightly. Strong et al. (2016) found a 12% increase when treatment was started at 10 months; in the current study, the 90th percentile lifespan increased 7% when treatment was started at 16 months (Table 1). Thus, 17aE2 joins rapamycin as an intervention effective later in life, although 17aE2 has no benefit for females. 17aE2, like rapamycin, works just as well in males even though started in middle age (16 months) and almost as well when started as late as 20 months. Acarbose is a bit different: started in middle age, it still works, but only about ½ as well as it does when it starts in young mice (Harrison et al., 2019).

The dramatic sexual dimorphism in response to 17aE2 treatment may help elucidate aspects of the mechanism by which sex hormones regulate lifespan. In UM‐HET3 mice, diet restriction (DR) increases male and female lifespans to a similar degree (Flurkey et al., 2010). This is not the case for 17aE2. Both male and female mice given 14.4 ppm 17aE2 diets starting at 10 months of age were 14%–18% lighter than controls at 12, 18, and 24 months yet lifespans only increased in males (Strong et al., 2016, Figure 1, Figure S1), implying that the male‐specific effects are not mediated by DR.

Rapamycin, like 17aE2, increases lifespan when treatment is initiated at 20 months of age; however, unlike 17aE2, rapamycin benefits both male and female mice (Harrison et al., 2009, 2014; Miller et al., 2011, 2014). Thus, the male‐specific lifespan benefit of 17aE2 contrasts with other treatments that produce comparable benefits in both sexes, making this model valuable for investigation of sexually dimorphic, and potentially novel, mechanisms of aging.

Garratt et al. (2017, 2018) evaluated effects of 17aE2 feeding on metabolic parameters in mature adult UM‐HET3 mice of both sexes. In mice started on 17aE2 at 4 months of age, and euthanized at 12 months of age, only intact (i.e., non‐castrated) males showed increased insulin sensitivity and improved glucose tolerance, enhanced hepatic mTORC2 signaling, increased Akt activity, and phosphorylation of FOXO1a. Females and castrated males did not show these changes, while ovariectomized females showed some of the benefits. Using mice treated at the same ages, Garratt et al. (2018) found changes in liver amino acids, and urea cycling, in untargeted metabolomic analyses; these changes also were specific to intact males. If testicular hormones play an important role in lifespan extension when 17aE2 is given at 10–20 months of age, perhaps the reduction in benefits observed between 16 and 20 months is partly caused by a decline in androgen with age in male mice (Coquelin & Desjardins, 1982). Only males (Garratt et al., 2018) had elevated levels of two conjugated estriols when given 17aE2. This suggests that males, but not females, metabolize 17aE2 into one or more estriol derivatives and that the sex‐specific beneficial effects may be due to an estriol derivative rather than to 17aE2 per se. The ITP is testing this hypothesis by comparing lifespans in male and female mice given diets containing estriol.

Strong et al. (2016) noted that 17aE2 at the standard 14.4 ppm dose reduced body weights in females and altered uterine weights in ovariectomized mice. Thus, 17aE2 is partially active in females, so the absence of an effect on female lifespan is not because bioavailability is entirely absent. These observations support the idea that testicular hormones play an important role in lifespan extension by 17aE2. Interestingly, the wide range of cancers that cause most deaths in UM‐HET3 mice (Harrison et al., 2014; Lipman et al., 2004; Miller & Chrisp, 2002) most likely are postponed by 17aE2 to increase lifespan in males.

Stout et al. (2017) reported that 14.4 ppm 17aE2 alleviates age‐related metabolic and inflammatory dysfunction in 18‐ or 20‐month‐old male C57BL/6 mice treated for 10 or 15 weeks. Treated males showed reduced energy intake but not expenditure, and reduced inflammation, fat, glucose, insulin, and mTOR complex 1, while increasing AMPKα. Miller, Pharaoh, et al. (2020) compared effects of short‐term 18% diet restriction (DR) and dietary 17aE2 in 20‐month‐old male C57BL/6 mice. The 17aE2 had no effect on protein‐to‐DNA synthesis rates compared with controls in any tissue, while 18% DR produced the large changes in protein‐to‐DNA synthesis rates expected with lifelong 40% DR. Using RNA‐seq, Tyshkovskiy et al. (2019) found feminizing effects with most of the anti‐aging candidate drugs they tested, increasing RNAs characteristic of females and decreasing RNAs characteristic of males. Interestingly, the exception was 17aE2. It might be informative to feed 17aE2 to young or middle‐aged mice and see how quickly the RNA profile is modified to resemble that seen in mice given 17aE2 for most of their lives.

None of the other four drugs tested in the 2015 cohort, that is, NR, CC, GGA, and MIF098, led to lifespan improvement in either sex. As reviewed in the introduction, in many species, total NAD levels decline with age, increasing NAD levels benefits many physiological systems in mice and men, and a small increase in mouse lifespan was reported. Our finding that NR has no effect on lifespan in the genetically variable mice that best model the human population is thus a surprise. In fact, the two points of most interest in this paper are that 17aE2 is effective when given later in life, and that NR has no effect on lifespan.

Each drug was detectable in food pellets, and metabolites of NR and CC were detected in plasma of treated mice. While it is possible that one or more of these drugs might have led to health benefits if used at a different dose, or in another stock of mice, or if started or stopped at a different age, the most plausible interpretation is that none of the drugs slows aging or prevents disease in a genetically heterogeneous mouse population.

Lifespan studies of the ITP demonstrate that, when following precise standards, mouse lifespan can be reproducibly extended by drugs in the diet (Harrison et al., 2009, 2014, 2019; Miller et al., 2011, 2014; Miller, Harrison, et al., 2020; Strong et al., 2008, 2016). Positive results are important, as showing here that 17aE2 is beneficial when started later in life. But negative results are also important, as showing here that NR failed to increase lifespan.

## 4 EXPERIMENTAL PROCEDURES

### 4.1 Mouse production, maintenance, and estimation of lifespan

**FIGURE 4:**
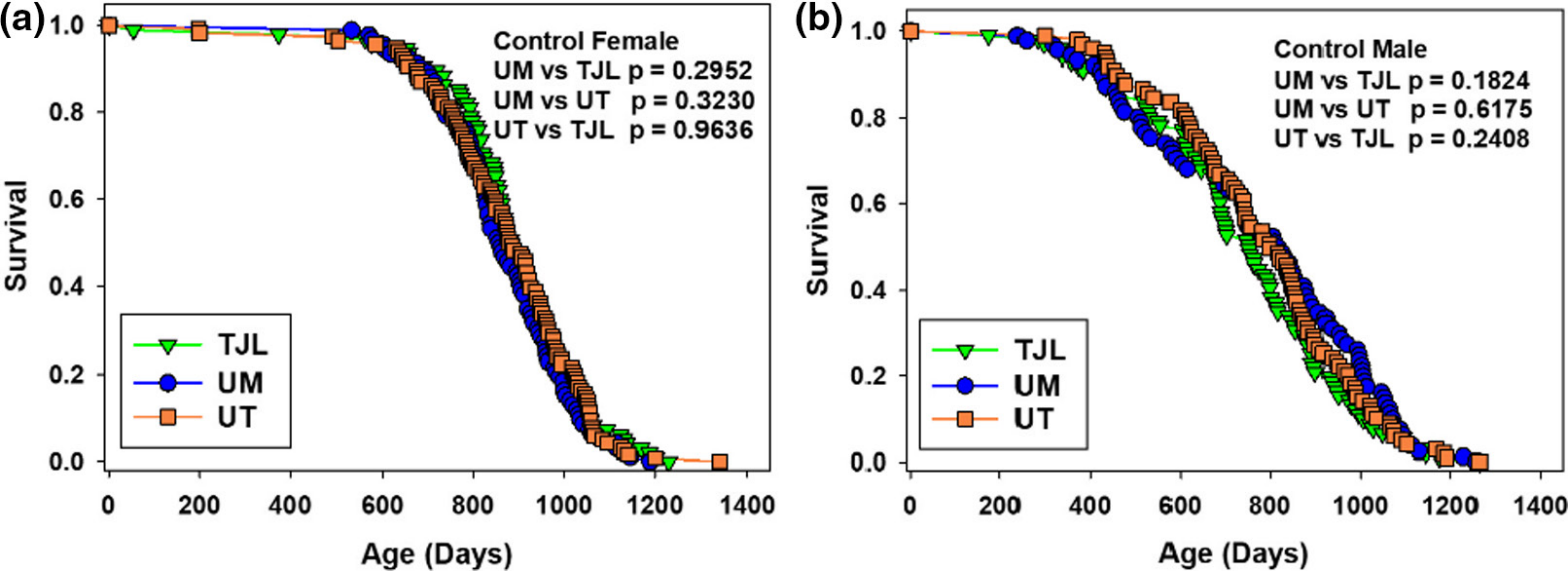
Effects of site on control lifespans in this cohort. Panel A shows females, and Panel B shows males. *p* values are for median lifespans; controls did not differ significantly among the sites

UM‐HET3 mice were produced at each of the three test sites as previously described (Harrison et al., 2009; Miller et al., 2011; Strong et al., 2008), where environmental conditions are presented in detail. The dams of the test mice were CByB6F1/J, TJL stock #100009 (dams, BALB/cByJ; sires, C57BL/6 J). The sires of the test mice were C3D2F1/J, TJL stock #100004 (dams, C3H/HeJ; sires, DBA/2 J). In each site, breeding mice were fed LabDiet^®^ 5008 mouse chow (PMI Nutritional International, Bentwood, MO). As soon as mice were weaned, they were fed LabDiet^®^ 5LG6 distributed to the test sites from the same batch. Males were initially housed 3 per cage, while females were housed 4 per cage; numbers per cage declined as mice died. Figure 4 shows that lifespans for both female and male controls were similar at all 3 sites.

Details of the methods used for health monitoring were provided previously (Harrison et al., 2009; Miller et al., 2011; Strong et al., 2008). In brief, each of the three colonies was evaluated four to twelve times each year for infectious agents. All such surveillance tests were negative for pathogens at all three sites throughout the entire study period.

### 4.2 Removal of mice from the longevity population

Mice were removed from the study because of fighting or accidental death (e.g., during chip implantation) or chip failure, or because they were used for another experimental purpose. For log‐rank survival analyses, all such mice were “censored,” that is, treated as alive at the date of their removal from the protocol and lost to follow‐up thereafter. These mice were not included in calculations of median longevity. For the mice in Table 1, % males removed: control, 9%; 17aE2‐16, 3%; 17aE2‐20, 10%; NR, 7%; CC, 12%; GGA, 13%; and MIF098, 6%. Most of these males were removed because of fighting. Fewer females were removed: control, 0.3%; NR, 1.5%; CC, 3%; GGA, 0; and MIF098, 0.

Thus, mice removed from the longevity population were included in log‐rank test calculations. The question as to whether removal is random does not apply, since no mice were removed for other purposes. The statistical approach used is the usual Kaplan–Meier calculation, based on the log‐rank test, in which mice that do not die a natural death (typically because of removal for humane reasons) are treated as known to be alive at the date of removal, with unknown date of death.

### 4.3 Estimation of age at death (lifespan)

At UM and UT, mice were examined daily for signs of ill health from the time they were set up in the experiment. At TJL, mice over 500 days of age were examined daily and twice a day once they were marked as ill. Mice were euthanized for humane reasons if so severely moribund that they were considered, by an experienced technician, unlikely to survive for more than an additional 48 hours. TJL’s definitive endpoint criterion is the non‐responsiveness of a mouse to being touched, and which is usually accompanied by one or more of the following: slow respiration, feeling cold to the touch, a hunched‐up appearance with matted fur, signs of sudden weight loss, failure to eat and drink, prominent appearing ribs and spine, and sunken hips. The age at which a moribund mouse was euthanized was taken as the best available estimate of its natural lifespan. Mice found dead were also noted at each daily inspection, giving the lifespan.

### 4.4 Control and experimental diets

TestDiet^®^, Inc., a division of Purina Mills (Richmond, IN), prepared batches of radiation‐sterilized LabDiet^®^ 5LG6 food which were ground to powder, and to which each test substance was added, as well as control diets from the same large batch treated the same way except that no drugs were added when diets were ground and mixed. These were prepared at intervals of approximately 4 months, and TestDiet^®^ shipped food from the same batch to each of the three sites. For the interventions in this paper, except E2, the doses used were suggested by the experts who proposed the compound and were tested in pilot studies to be sure that the mice were not harmed over 8 weeks.

1. 17‐α‐estradiol (17aE2) was purchased from Steraloids Inc. (Newport, RI, USA) and mixed at a dose of 14.4 milligrams per kilogram diet (14.4 ppm). Male mice were fed the 17aE2 diet continuously starting at either 16 or 20 months of age.
2. Nicotinamide riboside (NR) was generously provided by ChromaDex, 1751 S Fordham Street, Ste:350, Longmont, CO 80503, as the chloride salt, and mixed at a dose of 1,000 mg NR Chloride per kilogram diet (1,000 ppm). Mice were fed the NR diet continuously starting at 8 months of age. Upon assay, the amounts of NR plus nicotinamide totaled 878 ppm. For assay, control diet or plasma was spiked with known concentrations of NR and nicotinamide and compared with unknowns in the LC/MS/MS using mitoquinone D‐15 as the internal standard. Assaying four different batches of diet pellets, amounts of nicotinamide +NR in ppm were as follows: 196 + 393 = 589 (67% of the expected level); 371 + 414 = 785 (89% of expected); 101 + 551 = 652 (74% of expected); and 241 + 453 = 695 (79% of expected).
3. Candesartan cilexetil (CC) and candesartan were purchased from TOCRIS Bioscience (Bristol, United Kingdom); CC was mixed at a dose of 30 mg per kilogram diet (30 ppm). Mice were fed the CC diet continuously starting at 8 months of age. CC and candesartan were quantified mixing calibrator samples and unknowns with 10 µL of 100 µg/mL withaferin A and 1 ml of mobile phase B, and assayed in the LC/MS/MS. The ratios of CC and candesartan peak areas to withaferin A peak areas for each unknown sample were compared against the ratios obtained by the calibration samples to quantify CC and candesartan. Nine separate batches of diet pellets were tested, with an average of 84% of the expected level (range: 58–122%).
4. Geranylgeranylacetone (GGA) was purchased from Sigma‐Aldrich Chemical Company (St. Louis, MO) and mixed at a dose of 600 mg per kilogram diet (600 ppm). Mice were fed the GGA diet continuously starting at 9 months of age. GGA was quantified using mixtures of known calibrators and unknown samples in the HPLC/UV. The peak area ratios for each unknown sample were compared against calibrator peak area ratios to quantify GGA. A similar method was described by Ding et al. (2007). Nine separate batches of diet pellets were tested with an average of 73% of expected amounts of GGA (range: 16%–123%).
5. MIF098 was obtained from Cheminpharma (Woodbridge, CT) and mixed at a dose of 240 mg per kilogram diet (240 ppm). Mice were fed the MIF098 diet continuously starting at 8 months of age. It was measured using spiked controls and injecting well‐mixed standard amounts of calibrator and unknown in the HPLC/UV. Ratios of MIF 098 peak areas were compared to clonazepam peak area standards. In 9 different batches of diet pellets, the % of expected amounts of MIF098 averaged 94% and ranged from 74% to 112%.

The 2015 cohort of UM‐HET3 mice was also used to test the effects of canagliflozin, an inhibitor of SGLT2 (Miller, Harrison, et al., 2020).

### 4.5 Statistical methods

Significance tests about survival effects are based upon the two‐tailed log‐rank test at *p* < 0.05, stratified by test site, with censored mice included up until their date of removal from the longevity population. Data from male and female mice are considered separately. In statistical tests described in the text, *P* values are two‐tailed and reported without adjustment for multiple comparisons. Statistical claims related to maximum lifespan are based on Wang et al. (2004), using the Fisher exact test to compare the proportions of surviving mice, in control and test groups, at the age corresponding to the 90th percentile for survival in the joint distribution of the control and test groups. For the pooled data sets, surviving mice were enumerated at the 90th percentile age for each site separately, and these counts were combined for the overall Fisher exact test.

The raw data used here are available at the Mouse Phenome Database https://phenome.jax.org/projects/ITP1, The Jackson Laboratory, Bar Harbor, ME. This is supported by the Mouse Phenome Project and is a public data repository that provides the authoritative source for the raw and summary data from the ITP, along with visualizations for exploration of lifespan and related phenotype data.

## CONFLICT OF INTEREST

None.

## AUTHOR CONTRIBUTIONS

DEH, RS, and RAM are the principal investigators at the three collaborating institutions and are responsible for project design, supervision of technical personnel, interpretation of results, and preparation of manuscript drafts. MAJ and MLC supervised the Pharmacology Core and helped with the manuscript; JFN and KF advised on experimental design and interpretation and helped with the manuscript. NK, AM and AB did NR experiments. Laboratory manager PR supervised laboratory procedures and data collection at The Jackson Laboratory site, and organized diet preparations for all three sites. MB and TMS analyzed data and organized the database. FM served as the project officer for the National Institute on Aging and contributed to program development, experimental design, and analysis. EF supervised laboratory personnel and data collection at the UTHSCSA site. ALS proposed NR; GC proposed CC; KK proposed GGA; RB and RM proposed MIF‐098; and LL prepared it.

## Data Availability

Mouse Phenome Database https://phenome.jax.org/projects/ITP1, The Jackson Laboratory, Bar Harbor, ME.
