# Corrigendum to: 17‐a‐estradiol late in life extends lifespan in aging UM‐HET3 male mice; nicotinamide riboside and three other drugs do not affect lifespan in either sex

**DOI:** 10.1111/acel.13672

**Published:** 2022-07-27

David E. Harrison, Randy Strong, Peter Reifsnyder, Navasuja Kumar, Elizabeth Fernandez, Kevin Flurkey, Martin A. Javors, Marisa Lopez‐Cruzan, Francesca Macchiarini, James F. Nelson, Adrian Markewych, Alessandro Bitto, Amy L. Sindler, Gino Cortopassi, Kylie Kavanagh, Lin Leng, Richard Bucala, Nadia Rosenthal, Adam Salmon, Timothy M. Stearns, Molly Bogue, Richard A. Miller. *Aging Cell*. 2021;20:e13328. https://doi.org/10.1111/acel.13328

In the original published article, the authorship list was not complete. Adrian Markewych should have been added as the 11^th^ author with the affiliation Department of Pathology, University of Washington Medical Center, Seattle, WA, USA.

The article has been corrected online.

The authors regret the error.

